# The impact of integrated urban and rural resident basic medical insurance on health service equity: Evidence from China

**DOI:** 10.3389/fpubh.2023.1106166

**Published:** 2023-03-13

**Authors:** Qiaosheng Li, Lanyue Zhang, Weiyan Jian

**Affiliations:** Department of Health Policy and Management, School of Public Health, Peking University, Beijing, China

**Keywords:** health insurance integration, URRBMI, health service utilization, health equity, China

## Abstract

**Background:**

Many countries and regions worldwide are improving their healthcare systems through the integration and unification of health insurance programs covering different groups of people. In China, the past 10 years has been the time when Chinese government promote the Urban and Rural Residents Basic Medical Insurance (URRBMI) by integrating the Urban Residents' Basic Medical Insurance (URBMI) and New Rural Cooperative Medical Scheme (NRCMS).

**Objectives:**

To evaluate the impact of the URRBMI on equity in relation to health services.

**Methods:**

The quantitative data used in this study were obtained from the CFPS 2014–2020 database, and all respondents with health insurance type UEBMI, URBMI, and NRCMS were included. UEBMI respondents were set as the control group and URBMI or NRCMS as the intervention group, and a DID method model was used to analyze the impact of integrating health insurance on health service utilization, costs and health status. Heterogeneity analysis was also conducted after stratifying the sample according to income level and chronic disease status. This was done to investigate whether there were differences in the effects of the integrated health insurance program across different social groups.

**Results:**

The implementation of URRBMI is found to be associated with a significant increase in inpatient service utilization (OR = 1.51, *P* < 0.01) among rural Chinese residents. Regression results by income stratum show that the utilization of inpatient services increased in rural areas for high-, middle- and low-income groups, with the fastest increase (OR = 1.78, *P* < 0.05) emerging for low-income groups. Analysis by chronic disease status shows that rural residents with chronic disease are associated with a higher increase in hospitalization rates (OR = 1.64, *P* < 0.01).

**Conclusion:**

The implementation of URRBMI is found to have improved health insurance's ability to withstand risks and effectively improve access to health services for rural residents. In this regard, it can be considered as playing a positive role in bridging the gap in health service utilization between rural and urban areas and in improving regional equity.

## 1. Introduction

One of the critical dimensions of universal health coverage (UHC) most widely advocated worldwide is equitable access to health services. If equitable access to health services holds in respect of a given region, then people of different socioeconomic status have equal opportunities to access health services when they are needed. Among the many factors influencing access to health care, the coverage and level of coverage of health insurance plans are critical (1). Based on the “law of large numbers”, health insurance systems rely on a variety of sources to raise funds and provide compensation for a specific range of health services to assist those in need. This is done through a risk-sharing mechanism, which plays an active role in preventing catastrophic health expenditures and ensuring that health service needs can be met.

Since 2003, China has witnessed the establishing of three government-led health insurance schemes: the “Urban Employees' Basic Medical Insurance” (UEBMI), covering employees in cities; the “Urban Residents' Basic Medical Insurance” (URBMI), covering the urban non-working population; and the “New Rural Cooperative Medical Scheme” (NRCMS), applying to all rural residents (2). This health insurance system, with its multitude of schemes, has emerged as suffering from certain limitations, particularly in terms of fund raising, in respect of which it can be observed that UEBMI has the highest fundraising capacity and NRCMS the lowest (3, 4). In 2013, for example, per capita funding for each of the three types of health insurance scheme were 1561 CNY, 400 CNY and 370 CNY (Ministry of Human Resources and Social Security of the People's Republic of China, 2013; National Health Commission of the People's Republic of China, 2013), and such gaps in fundraising capacity can be observed to have an impact on benefits packages. NRCMS has the highest ability of cost sharing of these three health insurance schemes (5–7). According to the National Health Service Survey (NHSS) in 2013, the co-payment rates for NRCMS, URBMI and UEBMI were 49.9, 46.4, and 31.2%, respectively (8).

Such disparities in co-payment rate leads to a significant gap in health service utilization between the enrollees on URBMI and NRCMS and those on UEBMI. Based on NHSS, in 2013, the inpatient rate for UEBMI enrollees was 11.2%, and the non-hospitalization rate due to financial hardship was 4.6% (8). However, the inpatient rate for URBMI enrollees was 7.1% and for NRCMS it was 9.0%, and the non-hospitalization rates for URBMI and NRCMS were 9.1 and 8.2%, respectively. This highlights that differences in fundraising levels result in differences in fund pool size, which affects the co-payment rate, and further influences the disparity in health services utilization among enrollees of different health insurance schemes, as well as exacerbating the inequity in health services between urban and rural areas.

In 2009, lacking a rapid increase in the resources to finance such healthcare schemes, the Chinese government began to strengthen the capacity of risk resistance of health insurance schemes by expanding the risk pool through integration of URBMI and NRCMS (9). This program of integration was named “Urban and Rural Resident Basic Medical Insurance” (URRBMI). The reasoning underpinning this move was that, even if funding would not increase significantly, having a large risk pool would improve benefit packages to a useful extent (7, 10).

The NRCMS can be observed as being a package with a lower level of benefits than the URBMI. And URRBMI, as the health insurance integration plan, was associated with emphasizing bringing the NRCMS benefit package in line with the URBMI (i.e., upgrading the NRCMS benefit) as a means of assisting previously disadvantaged rural residents and improving their access to healthcare. Internationally, it can be seen that similar such methods of engaging in health insurance integration exists. For example, in 2011, Indonesia passed a law to merge all five existing government health insurance risk pools into one universal program in order to reduce inequalities in benefits by promoting cross-subsidization and decreasing administrative costs (11, 12). In addition, health financial equity has also been improved through health insurance integration in South Korea, Turkey and Thailand, where inequity in health financing has been addressed across different income and job groups, especially in rural areas (11).

China was able to initiate and fully implement URRBMI before the year of 2019. However, most of the most recent studies on the effect of integrating URBMI and NRCMS in the country are from before 2016. Moreover, such studies tend to be negatively affected by short implementation times and small sample sizes, rendering the results potentially unstable. It is on this basis that this study expands the timeframe to 2020 and uses the nationally representative data to assess the impacts of the integrated health insurance programs on health equity in China, obtaining more stable and reliable policy evaluation results through greater sample size analysis.

## 2. Study design and methods

### 2.1. Material

This study considers data from China Family Panel Studies (CFPS), which is a nationally representative, comprehensive, longitudinal social survey of the Chinese population. The database covers a wide range of topics and includes intergraded modules relating to education, regions, health and other areas of information (13, 14). During the sampling phase of the CFPS, a multi-stage random sampling method was used to select ~15,000 households nationwide. This involved interviewing all household members in each sample to obtain data using a face-to-face questionnaire (15). Strict quality control protocols are in force in respect of the project implementation process, including professional database construction. CFPS surveys are conducted every 2 years, with the most recent once having been conducted in 2020. CFPS is given ethical approval by the Biomedical Ethics Review Committee of Peking University, with all participants being required to provide written informed consent. The ethical approval number is IRB00001052-14010.

### 2.2. Sample

The data collection was conducted between 2014 and 2020, and the inclusion criteria for the sample was phrased in terms of all the individuals who participated in the CFPS survey. Participants were excluded if they were not insured by NCMS, URBMI, UEBMI, or URRBMI or if the relevant data entries were missing control or outcome values.

### 2.3. Variables

#### 2.3.1. Outcome variables

The following variables were selected as outcome variables: (1) whether the individual had been hospitalized in the past year (1 = yes; 0 = no), relating to the utilization of inpatient services; (2) total hospitalization expenditure in the past year (if the inpatient care utilization in past year = 0, this variable is absent); (3) outpatient care utilization in past 2 weeks (1 = yes; 0 = no); (4) medical expenditure in the past year other than hospitalization, relating to the total expenditure associated with the given outpatient over the past year (if the outpatient care utilization in past year = 0, this variable is absent); (5) total out-of-pocket medical expenditure paid by individuals in the past year, reflecting personal burden; (6) self-assessed health status (if the answer to “what is your health status? “is “Excellent; very good or good”, then the value is 1, if the answer is “Fair or Poor”, then the value is 0). All cost indicators were measured in RMB, and the consumer price index (CPI) level from the same survey period was used for standardization.

#### 2.3.2. Control variables

Social economic status variables were used as control variables, including the participants' personal characteristics, health status, location, etc., and the manner evident in the classification of these variables in the existing literature (16, 17). The specific rules governing this classification are provided in Table 1. The average levels of each control variable in the intervention group and the control group are close, indicating that the sample distribution in the two groups is relatively balanced.

**Table 1:** Definition and assignment of rules regarding independent variables.

**Variables**	**The form entered the research**	**Mean (intervention group)**	**Mean (control group)**
Sex	=0 female; =1 male	0.50	0.55
Age	Years old	47.86	46.22
Marital status	=0 with spouse (married, cohabiting); =1 no spouse (unmarried, divorced, widowed)	0.71	0.70
Chronic diseases	=0 no; =1 yes	0.17	0.18
Education level	=1 lower education (literate, kindergarten, elementary school); =2 secondary education (junior high school, high school); =3 higher education (college, bachelor's degree, master's degree, doctorate)	1.16	1.66
Self-assessed income level	=1 low; =2 middle; =3 high	1.68	1.73
Self-assessed social status level	=1 low; =2 middle; =3 high	2.02	1.87
Province	Contains all national codes of the provinces involved in the survey	–	–
Year	2014; 2016; 2018; 2020	–	–

### 2.4. Statistical analysis

**Figure 1:**
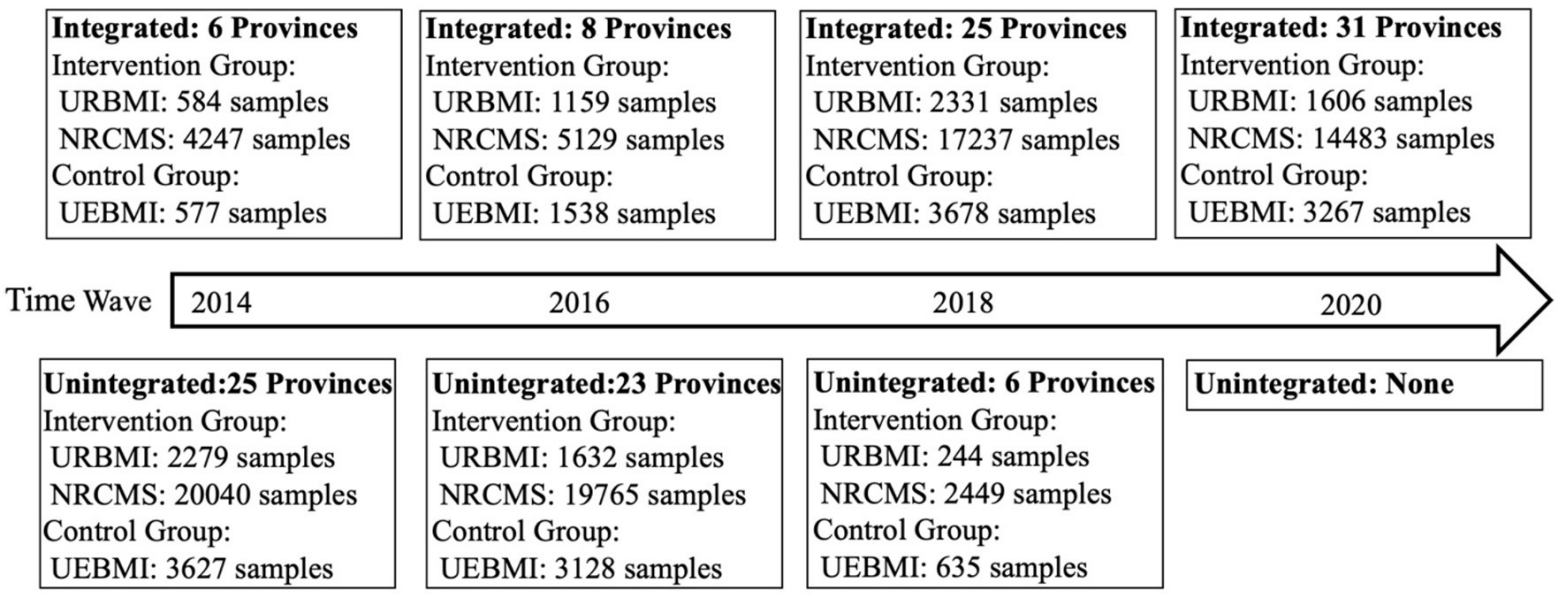
Intervention and control groups and waves.

The participants with UEBMI were defined as control group, while the participants with URBMI or NRCMS were defined as the intervention group. The samples of intervention and control groups in each wave before integration and after integration were clearly shown in Figure 1. A difference-in-difference approach (in short, when the dependent variable is a dichotomous variable, the logit model is adopted for regression; when the dependent variable is a continuous numerical variable, the log-linear model is adopted) was used for analyzing the impact of integrated medical insurance for urban and rural residents in terms of health services utilization and health status.

**Formula 1** shows the model settings regarding the impact of integration program on health service utilization and health status:


$$\begin{array}{l} Y_{ijt}=\lambda_{0}+\beta_{1}^{*}integrated_{jt}{+\beta }_{2}^{*}treat_{i}+\beta_{3}^{*}treat_{i}^{*}integrated_{jt}\\ \;+\delta^{*}x_{ijt}+{\alpha_{j}+\theta_{t}+\varepsilon }_{ijt}\; \end{array}$$

In **Formula (1)**, *i, j, t* represents survey participants, provinces and years, respectively. *Y*_*ijt*_ is the outcome variable, and *treat*_*i*_ is the dummy variable denoting the intervention group and control group (equaling 1 in the case of participants with URBMI or NRCMS, and 0 in the case of participants with UEBMI). *integrated*_*jt*_ is the dummy variable, equaling 1 if the province *j* has implemented integration in time *t*, and 0 otherwise. β_1_ represents the differences before and after the implementation of the integration, while β_2_ represents the differences between control group and intervention group. The coefficient β_3_ of the interaction term $treat_{i}^{*}integrated_{jt}$ is the real impact of integration implementation. *x*_*ijt*_ represents independent variables that need to be controlled. Fixed effects are selected in this model, α_*j*_ controls the fixed effects in different provinces, and θ_*t*_ controls the fixed effects in different years. ε_*ijt*_ is a random error term.

In addition, given that the impact of the implementation of integration program on health services utilization may relate to the income level and chronic disease status of the participants, heterogeneity analysis it was judged that also required to be conducted through stratification of income level and chronic disease status. The *event study* method was used to test the premise of the DID approach, that is, whether the trends of the outcome variables in control group and intervention group were parallel before health insurance integration by controlling all independent variables (with the last period before health insurance integration being taken as the benchmark). The model was set as **Formula (2)**:


$$\begin{array}{l} Y_{ijt}=\lambda_{0}+\overset{\sigma =6}{\underset{\sigma =1}{\sum }}\beta_{\sigma }^{*}1\left(T_{j}-t=\sigma \right)+\beta_{0}^{*}1\left(T_{j}=t\right)\\ \;+\overset{\tau =10}{\underset{\tau =1}{\sum }}\beta_{\tau }^{*}1\left(t-T_{j}=\tau \right)+\delta^{*}x_{ijt}+{\alpha_{j}+\theta_{t}+\varepsilon }_{ijt} \end{array}$$

In Formula (2), *T*_*j*_ is the integration time of province *j*, and 1(*T*_*j*_ − *t* = σ), 1(*T*_*j*_ = *t*), 1(*t* − *T*_*j*_ = τ) are dummy variables, representing the time before integration implementation, the time during the integration implementing, and the time after integration implementation, respectively.

The timeline of URRBMI implementation started in 2010, and the provinces data used in our study were from 2014 to 2020, which means we involved the data from URRBMI implementation 6 years ago (the provinces which started URRBMI in the year of 2020) to URRBMI implementation after 10 years (the provinces which started URRBMI in the year of 2010). The values of σ range from 1 to 6 while the values of τ range from 1 to 10, with the coefficient before this indicating the effect value of each period. Since CFPS surveys are conducted every 2 years, the coefficients are shown every 2 years. The meaning of the other variables are the same as in **Formula (1)**.

The statistical analysis in this study was carried out using Stata Version 16.0 (Stata/SE, StataCrop LLC, TX, USA).

## 3. Results

### 3.1. Sample characteristics

The characteristics of the sample in this study are summarized in Table 2. The total sample size was 109,635, with young adults (aged 18–40) and middle-aged adults (aged 41–60) being predominant in all of the included samples. The proportion of different genders reflected the ratio observed for the wider population. Nearly 80% of the participants in sample were married. Most of the participants have a low level of education, with <1% having a high level of education. Most of the participants have a medium level of income and social status, with relatively few having a low or high level. In addition, the majority of the participants are not listed as having a chronic disease. Aside from 2020, for which there is only a small sample, there were no significant differences in the sample size and composition features among the data for the other years considered (2012, 2014, 2016, and 2018). The proportion of URRBMI in sample increased year by year, increasing from 15.4% in 2014 to 83.1% in 2020 (as of 2020, all the provinces in CFPS database have completed health insurance integration, and the dates of URRBMI implementation in China are presented in Supplementary Table 1). The averages and standard deviations of the independent variables in this study are displayed in Table 3, classified by health insurance type.

**Table 2:** Sample characteristics.

	**2014**		**2016**		**2018**		**2020**	
	***N*** = **31,354**	**%**	***N*** = **32,351**	**%**	***N*** = **26,574**	**%**	***N*** = **19,356**	**%**
**Age**								
Below 18	719	2.3	714	2.2	509	1.9	591	3.1
18–40	11,414	36.4	11,877	36.7	9,112	34.3	7,027	36.3
41–60	12,193	38.9	11,928	36.9	10,334	38.9	7,361	38.0
61–80	6,355	20.3	7,048	21.8	6,303	23.7	3,981	20.6
Above 80	673	2.1	784	2.4	316	1.2	396	2.0
**Gender**								
Female	15,796	50.4	16,274	50.3	13,447	50.6	9,637	49.8
Male	15,558	49.6	16,077	49.7	13,127	49.4	9,719	50.2
**Marital status**								
No spouse	6,963	22.2	7,186	22.2	5,174	19.5	3,977	20.6
Have spouse	24,391	77.8	25,165	77.8	21,400	80.5	15,379	79.4
**Education level**								
Low	23,306	74.3	25,299	78.2	19,456	73.2	13,292	68.7
Middle	6,277	20.0	6,947	21.5	7,022	26.4	5,941	30.7
High	1,771	5.7	105	0.3	96	0.4	123	0.6
**Income level**								
Low	13,156	42.0	16,803	52.0	7,461	28.1	5,132	26.5
Middle	11,604	37.0	10,855	33.5	11,842	44.5	9,040	46.7
High	6,594	21.0	4,693	14.5	7,271	27.3	5,184	26.8
**Social status**								
Low	11,058	35.2	13,093	40.4	6,303	23.7	4,650	24.0
Middle	13,896	44.4	13,339	41.3	12,292	46.3	9,269	47.9
High	6,400	20.4	5,919	18.3	7,979	30.0	5,437	28.1
**Chronic disease**								
None	26,687	85.1	27,493	85.0	22,049	83.0	16,438	84.9
Yes	4,667	14.9	4,858	15.0	4,525	17.0	2,918	15.1
**Health insurance type** ^*^								
UEBMI	4,204	13.4	4,666	14.4	4,313	16.2	3,267	16.9
URBMI	2,279	7.3	1,632	5.0	244	0.9	0	0.0
NRCMS	20,040	63.9	19,765	61.1	2,449	9.2	0	0.0
URRBMI	4,831	15.4	6,288	19.5	19,568	73.7	16,089	83.1

* After the integration of URBMI and NRCMS, the unified medical scheme was named URRBMI.

**Table 3:** The average and standard deviation (SD) of dependent variables.

	**UEBMI**		**URBMI**		**NRCMS**	
	**Before integration**	**After integration**	**Before integration**	**After integration**	**Before integration**	**After integration**
Outpatient care utilization rate in past 2 weeks (%)	71.6	74.6	72.5	80.8	80.9	86.4
Annual outpatient expenditure (CNY)	2,846 (3,039)	2,785 (3,051)	2,293 (2,747)	2,615 (2,914)	1,884 (2,406)	2,093 (2,557)
Inpatient care utilization rate in past year(%)	11.2	10.5	9.2	11.5	8.6	12.7
Annual inpatient expenditure (CNY)	8,958 (16,968)	15,353 (20,037)	7,164 (13,796)	16,950 (22,720)	5,840 (11,521)	11,781 (18,496)
Annual out-of-pocket medical expenditure (CNY)	2,135 (6,320)	1,639 (5,669)	2,293 (7,226)	1,828 (6,623)	1,682 (5,091)	1,900 (6,477)
Self-assessed health rate (%)	75.0	77.5	67.4	69.3	69.3	67.3

### 3.2. Results of main regression

It can be seen from the *event study* (see Supplementary Figures 1–6) that 95% CI of the coefficients β of each dependent variable before health insurance integration contain 0, indicating that the difference between the intervention group and the control group is not statistically different. As a result, the trends of the outcome variables in control group and intervention group can be observed as being parallel before health insurance integration, and so DID approach can be used to analyze the policy effect.

From the main regression results presented in Table 4, it can be seen that, after the implementation of the integration, the odds ratio (OR) of inpatient rate of the participants is 1.47, which is statistically significant, while the changes in 2-week outpatient utilization, total expenditure of inpatient and outpatient, total expenditure of annual co-payment and health status are not statistically significant. Comparing the URBMI and NRCMS as the intervention group, respectively, with the control group (UEBMI), the integration program mainly produced significant effects among the participants whose original medical insurance type was NRCMS, and the odds ratio (OR) of inpatient rate among this group is 1.51. However, health service utilization and the expenditure associated with people who enrolled URBMI before the integration cannot be seen to have produced significant changes after the implementation of the integration program.

**Table 4:** The results of main regression.

**Model**	**Outpatient care utilization rate in past 2 weeks (OR)**	**Annual outpatient expenditure**	**Inpatient care utilization rate (OR)**	**Annual inpatient expenditure**	**Annual out-of-pocket medical expenditure**	**Self-assessed health rate (OR)**
	**Logit**	**Log-linear**	**Logit**	**Log-linear**	**Log-linear**	**Logit**
**Intervention group: URBMI &NRCMS**						
Treatment effects	1.1306	0.0823	1.4745^***^	0.0122	0.018	1.0046
Time trends	0.8650	−0.0547	0.7557^**^	−0.0672	−0.072	1.0611
Inter-group differences	1.2176	−0.327^***^	0.6689^***^	−0.224^***^	−0.232^***^	1.1750^***^
*R* ^2^	0.212	0.081	0.139	0.253	0.218	0.119
**Intervention group: URBMI**						
Treatment effects	1.1102	0.0365	1.1794	0.186	0.0308	1.1587
Time trends	0.8954	0.0843	0.9814	−0.103	−0.030	1.0463
Inter-group differences	0.9417	−0.396^***^	0.8294	−0.201^**^	−0.0951	0.9069
*R* ^2^	0.218	0.121	0.156	0.321	0.258	0.110
**Intervention group: NRCMS**						
Treatment effects	1.1222	0.0703	1.5141^***^	−0.0107	0.045	0.9891
Time trends	0.8523	−0.0532	0.7416^***^	−0.0642	−0.073	1.0610
Inter-group differences	1.3036^**^	−0.350^***^	0.6586^***^	−0.235^***^	−0.256^***^	1.2461^***^
*R* ^2^	0.213	0.080	0.140	0.243	0.216	0.120

*** *P* < 0.01,

** *P* < 0.05.

We used regression to determine the treatment effects based on difference-in-difference (DID) model between the intervention and control groups, controlling for samples' age, gender, marital status, education level, income level, social status, and chronic diseases.

### 3.3. Heterogeneity in results

The sample of this study was divided into three groups according to income (low income, middle income, high-income). Through regression analysis, it was found that the low-income group was more significantly affected by the integration, as evidenced by an odds ratio (OR) in inpatient service utilization of 2.70 for participants whose original insurance type was URBMI and one of 1.78 for participants whose original insurance type was NRCMS. Meanwhile, an increase of inpatient rate can also be witnessed for middle-or high-income participants whose original insurance type was NRCMS. In relation to this, the odds ratio (OR) of inpatient rate for middle-income participants is 1.53, and for high-income participant is 1.41 (see Table 5A).

**Table 5:** The results of heterogeneity analysis.

	**Outpatient care utilization rate in past 2 weeks (OR)**	**Annual outpatient expenditure**	**Inpatient care utilization rate (OR)**	**Annual inpatient expenditure**	**Annual out-of-pocket medical expenditure**	**Self-assessed health rate (OR)**
**Model**	**Logit**	**Log-linear**	**Logit**	**Log-linear**	**Log-linear**	**Logit**
**(A) Stratified by status in terms of income level**						
**INTERVENTION GROUP: URBMI**						
**High income**						
Treatment effects	0.9324	0.00620	1.0580	0.269	0.0102	1.3381
Time trends	1.0745	0.102	0.9188	−0.225	0.036	1.0187
Inter-group differences	1.0814	−0.476^***^	0.8124	−0.345^***^	−0.138	0.8070^*^
*R* ^2^	0.212	0.137	0.139	0.353	0.246	0.112
**Middle income**						
Treatment effects	0.2149	−0.0111	0.9554	0.000362	0.00634	0.9877
Time trends	0.8428	0.0648	0.9862	−0.0463	−0.0756	1.0667
Inter-group differences	0.9550	−0.236	0.9783	−0.0329	−0.0223	1.0387
*R* ^2^	0.233	0.134	0.154	0.410	0.261	0.103
**Low income**						
Treatment effects	2.3936	0.0640	2.7036^**^	−0.0211	−0.0338	2.2127
Time trends	0.7594	0.00266	1.1553	0.114	−0.0422	1.0273
Inter-group differences	0.3658^**^	−0.486	0.4848	0.151	−0.0291	0.9196
*R* ^2^	0.275	0.128	0.231	0.308	0.300	0.119
**INTERVENTION GROUP: NRCMS**						
**High income**						
Treatment effects	0.9457	−0.0072	1.4149^***^	0.1620	0.0627	1.0682
Time trends	0.8885	0.0060	0.7608	−0.1880	0.0133	1.0143
Inter-group differences	1.3882^**^	−0.2780^**^	0.7400^**^	−0.3030^***^	−0.1450^**^	1.1459
*R* ^2^	0.219	0.079	0.131	0.279	0.208	0.127
**Middle income**						
Treatment effects	1.1557	0.1980	1.5297^***^	−0.0985	0.0680	0.9217
Time trends	0.9569	−0.1350	0.7280	−0.0132	−0.1350	1.1036
Inter-group differences	1.3641^**^	−0.3870^***^	0.6456^***^	−0.1800^**^	−0.296^***^	1.3356^**^
*R* ^2^	0.204	0.097	0.135	0.268	0.219	0.109
**Low income**						
Treatment Effects	2.0319	−0.0192	1.7807^**^	−0.0332	0.1860	1.0418
Time trends	0.4792	−0.0995	0.6540	0.1030	−0.2030	1.0713
Inter-group differences	0.6894	−0.2200	0.5321^***^	0.0349	−0.413^***^	1.1998
*R* ^2^	0.243	0.064	0.167	0.152	0.207	0.107
**(B) Stratified by status in terms of chronic disease**						
**INTERVENTION GROUP: URBMI**						
**NCDs**						
Treatment effects	1.0786	0.0491	1.1494	0.0895	0.0182	1.3094
Time trends	1.0568	−0.0822	0.9230	0.0304	0.1170	1.0883
Inter-group differences	1.0462	−0.3020	0.8845	−0.1460	−0.0181	0.9970
*R* ^2^	0.224	0.065	0.114	0.266	0.145	0.093
**Non-NCDs**						
Treatment effects	1.1234	0.0153	1.2001	0.2130	0.0230	0.1530
Time trends	0.8372	0.2220	1.0050	−0.1710	−0.057	1.0517
Inter-group differences	0.9136	−0.4830^***^	0.8116	−0.225^**^	−0.127	0.8984
*R* ^2^	0.156	0.064	0.062	0.445	0.168	0.061
**Intervention group:NRCMS**						
**NCDs**						
Treatment effects	1.9233	0.0386	1.6361^***^	−0.0524	0.0531	0.9473
Time trends	0.8593	−0.0371	0.7261^**^	−0.0607	0.0833	0.8899
Inter-group differences	1.5658	−0.3840^***^	0.6041^***^	−0.3030^***^	−0.2250^***^	1.3672
*R* ^2^	0.207	0.028	0.069	0.197	0.061	0.063
**Non-NCDs**						
Treatment effects	1.1608	0.0766	1.4423^***^	−0.0515	0.0310	0.9910
Time trends	0.8309	−0.0595	0.7453^**^	0.0098	−0.1020	1.0711
Inter-group differences	1.2608^**^	−0.233^**^	0.7043^***^	−0.1150	−0.2590^***^	1.2382^***^
*R* ^2^	0.163	0.043	0.055	0.284	0.144	0.069

*** *P* < 0.01,

** *P* < 0.05,

* *P* < 0.1.

We used regression to determine the treatment effects based on difference-in-difference (DID) model between the intervention and control groups, controlling for samples' age, gender, marital status, education level, income level, social status, and chronic diseases.

In addition, stratified by status in terms of chronic disease, the results show a statistically significant effect regarding the implementation of the integration on both rural residents with and without chronic disease, mainly in the form of an increase in inpatient services utilization. Meanwhile, the odds ratio (OR) of inpatient rate of rural participants with chronic disease is 1.64, which is higher than that for those without chronic disease (only 1.44; see Table 5B).

## 4. Discussion

In this study, the influence of URRBMI in China was analyzed by using 8 years of CFPS data, focusing on the changes in the health service utilization gaps between urban and rural residents. The period of 2014 to 2020 was considered on the basis that, during this 8-year period, the URRBMI was introduced or implemented in most of the Chinese provinces. Compared with previous studies that consider only certain provinces or shorter-term impacts, the evidence obtained in this study is more reliable.

The analysis demonstrated that rural residents in China are associated with a significant increase in inpatient service utilization (OR = 1.51, *P* < 0.01) after the implementation of URRBMI. The regression results by income stratum show that the inpatient service utilization among high-, middle- and low-income groups in rural area all increased. Meanwhile, analysis in terms of chronic disease status showed a significant increase in inpatient rate for rural residents. These findings enable acceptance of the basic hypothesis that the integration of URBMI and NRCMS has increase the insurance cover level of NRCMS, thus increasing the accessibility of inpatient services. Moreover, the participants whose original health insurance type was URBMI with a low level of income can also be seen to have benefited from the integration program, since the inpatient utilization has also increased in this group. This may be due to the point that it is possible to spread risk through expanding the size of the pool, thus providing vulnerable groups with greater access to health services.

Furthermore, while the utilization of inpatient services increased among rural residents, no such increase was found in respect of inpatient expenditure, and the increase in personal burden was also not found to be significant. This suggests that the URRBMI is an initiative program that has had benefits for poorer service users. In addition, no changes were found for outpatient service utilization among rural residents. There are two potential reasons for this. First, the baseline of outpatient services among rural residents was higher; second, health insurance programs in China have been designed to focus on covering expenditure for inpatient services rather than outpatient services, reducing the impact of URRBMI on outpatient services (2).

Overall, it was found that inpatient utilization has significantly increased among rural residents after the implementation of URRBMI, especially among the lower-income population. In this respect, the findings reported here are consistent with those reported by Huang et al., who analyzed data from the China Health and Retirement Longitudinal Study (CHARLS) for the period between 2011 and 2015. Similar comments apply to work by Paek et al., who analyzed data from the Health and Welfare survey database from after 2013 in respect of Thailand and found that the Universal Coverage Scheme highly increased inpatient utilization among the low-income group (16, 18). At the same time, however, a very mixed range of results has been cited in previous studies exploring the effects of URRBMI on outpatient utilization. For example, in research by Ma et al., Shi et al., and Su et al., it is reported that that the integration of URRBMI has significantly improved outpatient care utilization among middle-aged and elderly people in rural China; Huang et al. and Xu et al., by comparison, find no significant impact of the integration on rural residents' outpatient care utilization (16, 19–22). In the present study, a positive increase was found on outpatient utilization among low- and middle-income rural residents, but not to a statistically significant extent. This pattern may be explained in terms of the difference in datasets used. While previous studies have mainly focused on middle-aged and older residents or local situations, the present study used a nationally representative and longitudinal dataset covering the entire population to the inclusion of eight previous years of data (16, 19–22).

There are several limitations affecting the current study that must be acknowledged. First, due to certain constraints, the CFPS database lacks data on the co-payment rates of inpatient and outpatient services for participants at the individual or group level. As a result, it was not possible to evaluate insurance-related benefits after the implementation of URRBMI. Secondly, information is absent on the level of healthcare facilities utilized by participants, and so it was not possible to evaluate whether URRBMI caused changes in patient flow or fluctuations in health expenditure. Finally, it was not possible to determine whether patients have been overutilizing inpatient services in such a way that this has resulted in a “false” increase in inpatient rate after the increase of health care security level. This is due to a lack of specific information on inpatient conditions, including primary diagnoses and so on.

## 5. Conclusion

China URRBMI implementation has expanded the risk pool and improved the ability of the health insurance program to withstand risks. It has also improved coverage despite constraints in funding. In this respect, URRBMI has effectively improved health service utilization among rural residents, and this has played a positive role in narrowing the gaps in health service utilization between rural and urban areas, as well as in regional equity in health services. There is good reason to consider that such experiences can be extended to countries and regions where there are still multiple health insurance programs covering different populations. If this is done, it may be possible to improve the equity of health services by enhancing management capacity and seeking to integrate health insurance programs. In this way, progress toward UHC's goal of ensuring equitable access to health services can be made.

## Data availability statement

The datasets generated and analyzed for this study can be found in the CFPS repository. Please see http://www.isss.pku.edu.cn/cfps/ for more details, further inquiries can be directed to the corresponding author.

## Ethics statement

The studies involving human participants were reviewed and approved by the Biomedical Ethics Review Committee of Peking University approved CFPS, and all participants were required to provide written informed consent. The ethical approval number was IRB00001052-14010. Written informed consent to participate in this study was provided by the participants' legal guardian/next of kin.

## Author contributions

QL performed the data compilation and formal data analysis, interpreted the findings, and the manuscript. LZ conceptualized, drafted, wrote the manuscript, and interpreted the findings. WJ conceived of the study and participated in its design and coordination and helped to draft the manuscript. All authors have read and approved the final version of the manuscript and agree with the order of presentation of the authors.
