# Supplementary material for: The impact of integrated urban and rural resident basic medical insurance on health service equity: Evidence from China
Source: Front Public Health. 2023 Mar 13;11:1106166. doi: 10.3389/fpubh.2023.1106166 (PMC10040545; doi:10.3389/fpubh.2023.1106166)
Supplement: Supplementary file 1 [file Table_1.DOCX]

Supplementary Material

# Supplementary Tables

Appendix Table 1 Time of URRBMI implementation in China

| **Codes for Administrative Divisions** | **Province（Autonomous region/ municipality）** | **Year** |
| --- | --- | --- |
| 11 | Beijing | 2018 |
| 12 | Tianjin | 2010 |
| 13 | Hebei | 2017 |
| 14 | Shanxi | 2017 |
| 21 | Liaoning | 2020 |
| 22 | Jilin | 2019 |
| 23 | Heilongjiang | 2018 |
| 31 | Shanghai | 2016 |
| 32 | Jiangsu | 2018 |
| 33 | Zhejiang | 2014 |
| 34 | Anhui | 2017 |
| 35 | Fujian | 2016 |
| 36 | Jiangxi | 2017 |
| 37 | Shandong | 2014 |
| 41 | Henan | 2017 |
| 42 | Hubei | 2017 |
| 43 | Hunan | 2017 |
| 44 | Guangdong | 2012 |
| 45 | Guangxi | 2017 |
| 50 | Chongqing | 2009 |
| 51 | Sichuan | 2017 |
| 52 | Guizhou | 2020 |
| 53 | Yunnan | 2017 |
| 61 | Shaanxi | 2017 |
| 62 | Gansu | 2018 |
| Note: From the websites of the governments or health departments of all provinces (autonomous regions/ municipalities). | | |
