# Supplementary figures and images for: The impact of integrated urban and rural resident basic medical insurance on health service equity: Evidence from China
Source: Front Public Health. 2023 Mar 13;11:1106166. doi: 10.3389/fpubh.2023.1106166 (PMC10040545; doi:10.3389/fpubh.2023.1106166)

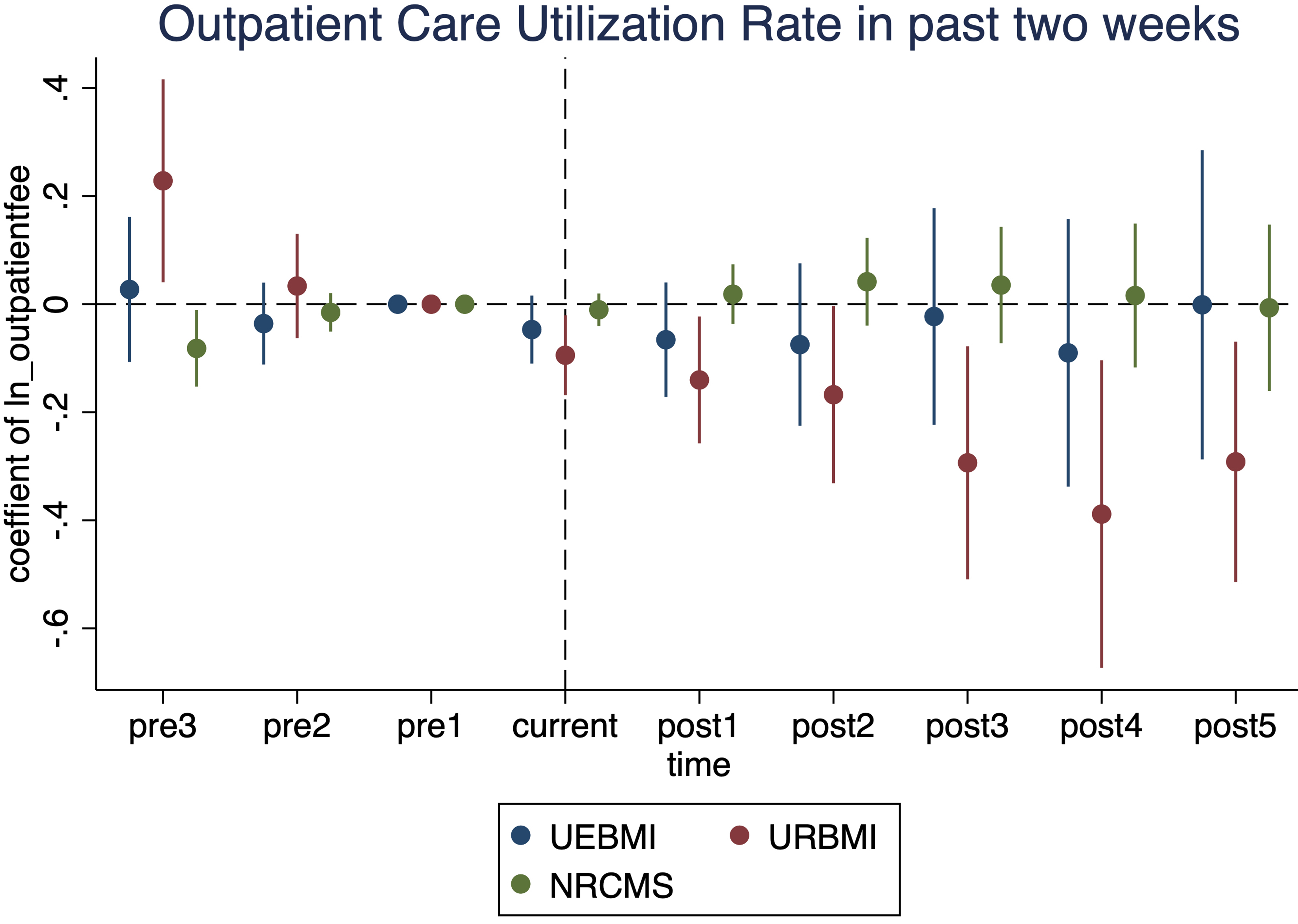

Supplement: Supplementary file 2 [file Image_1.JPEG]

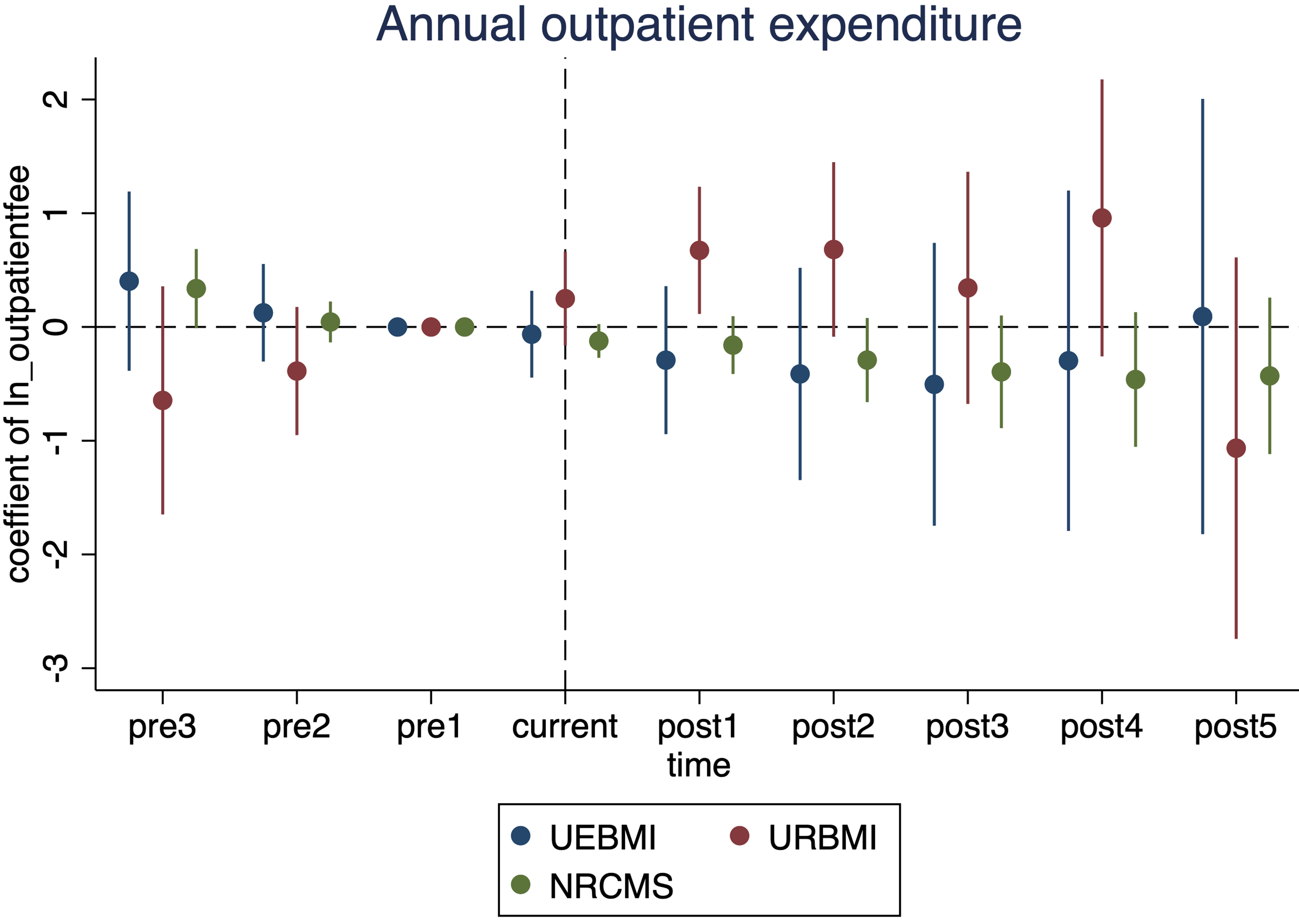

Supplement: Supplementary file 3 [file Image_2.JPEG]

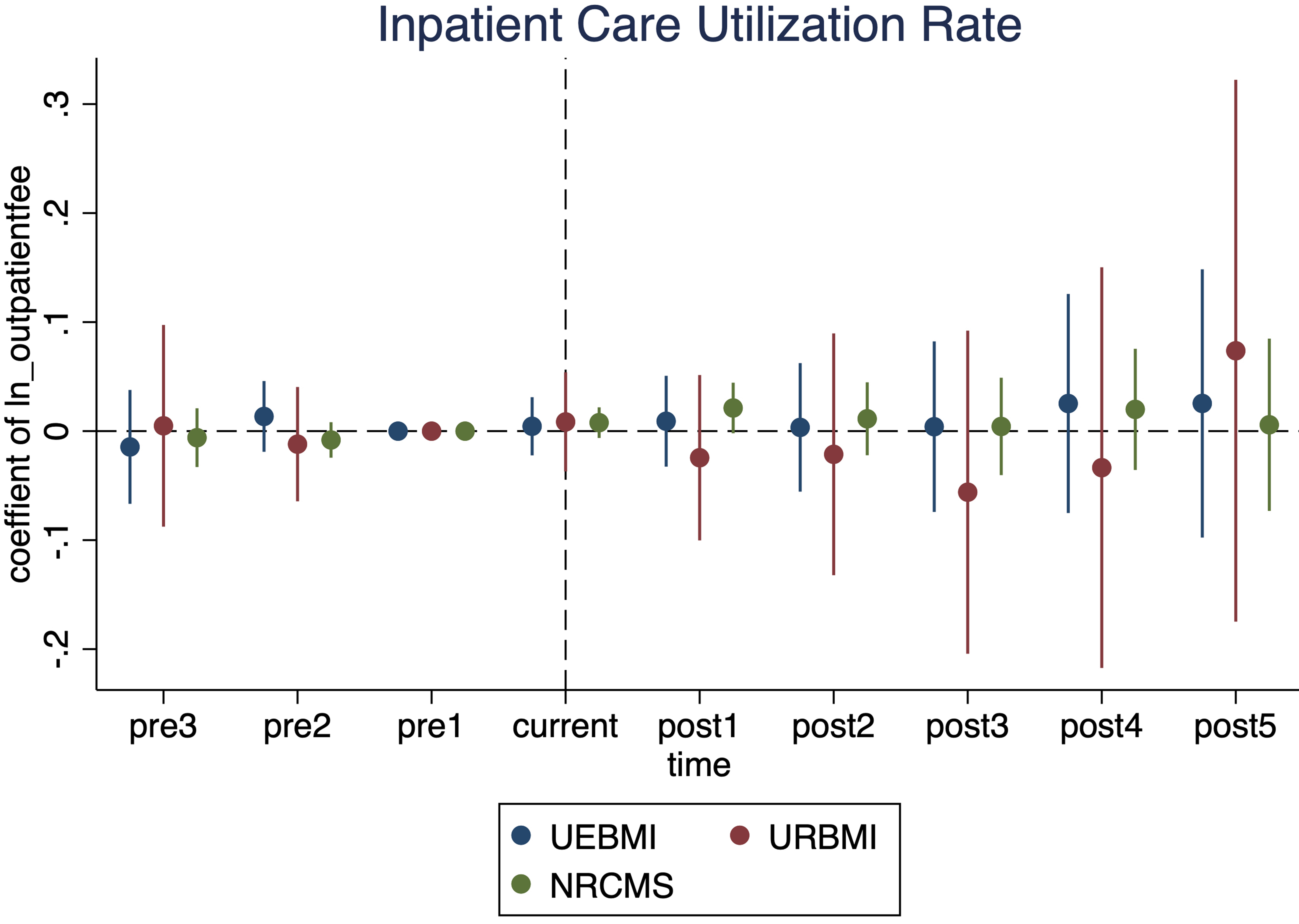

Supplement: Supplementary file 4 [file Image_3.JPEG]

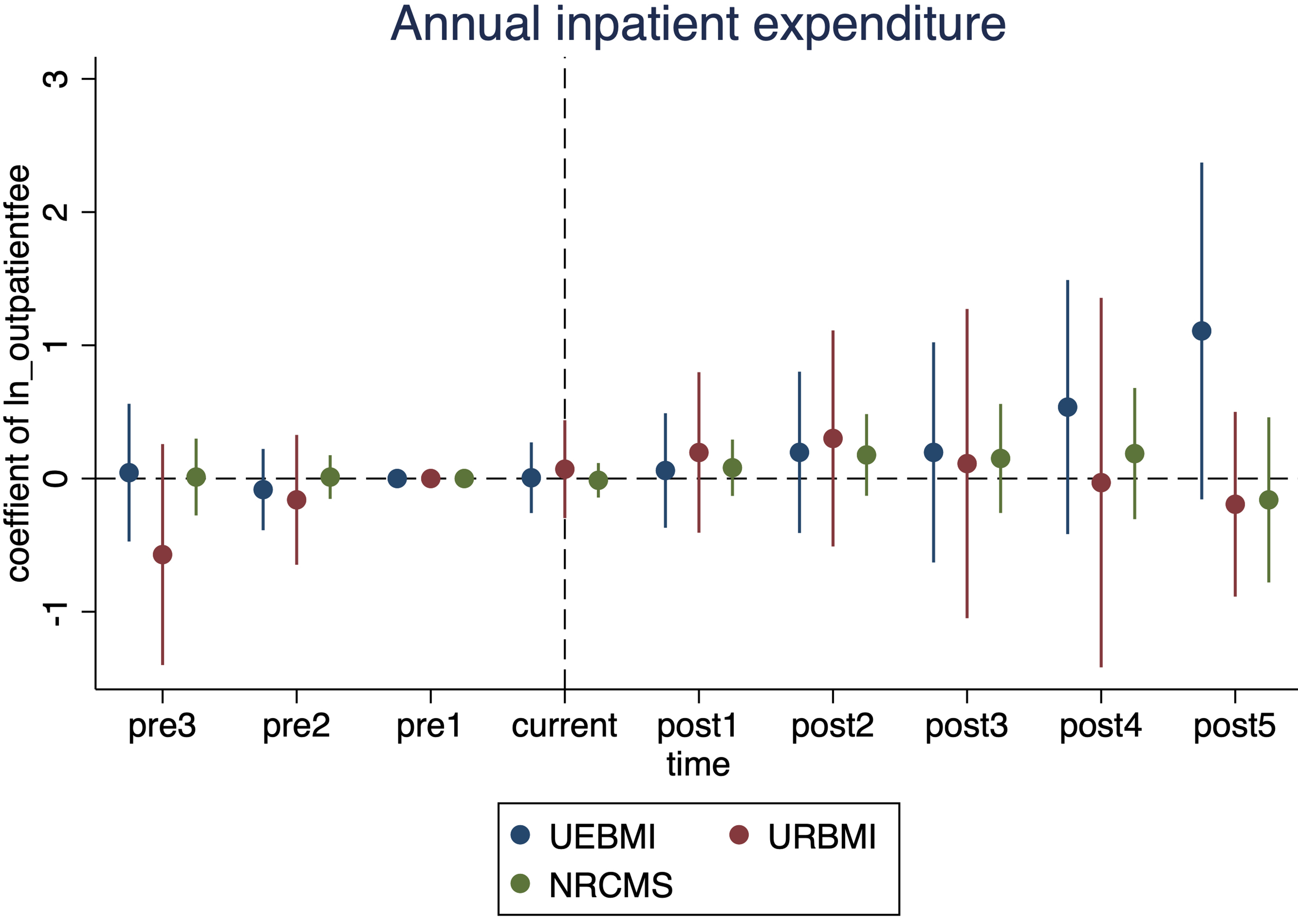

Supplement: Supplementary file 5 [file Image_4.JPEG]

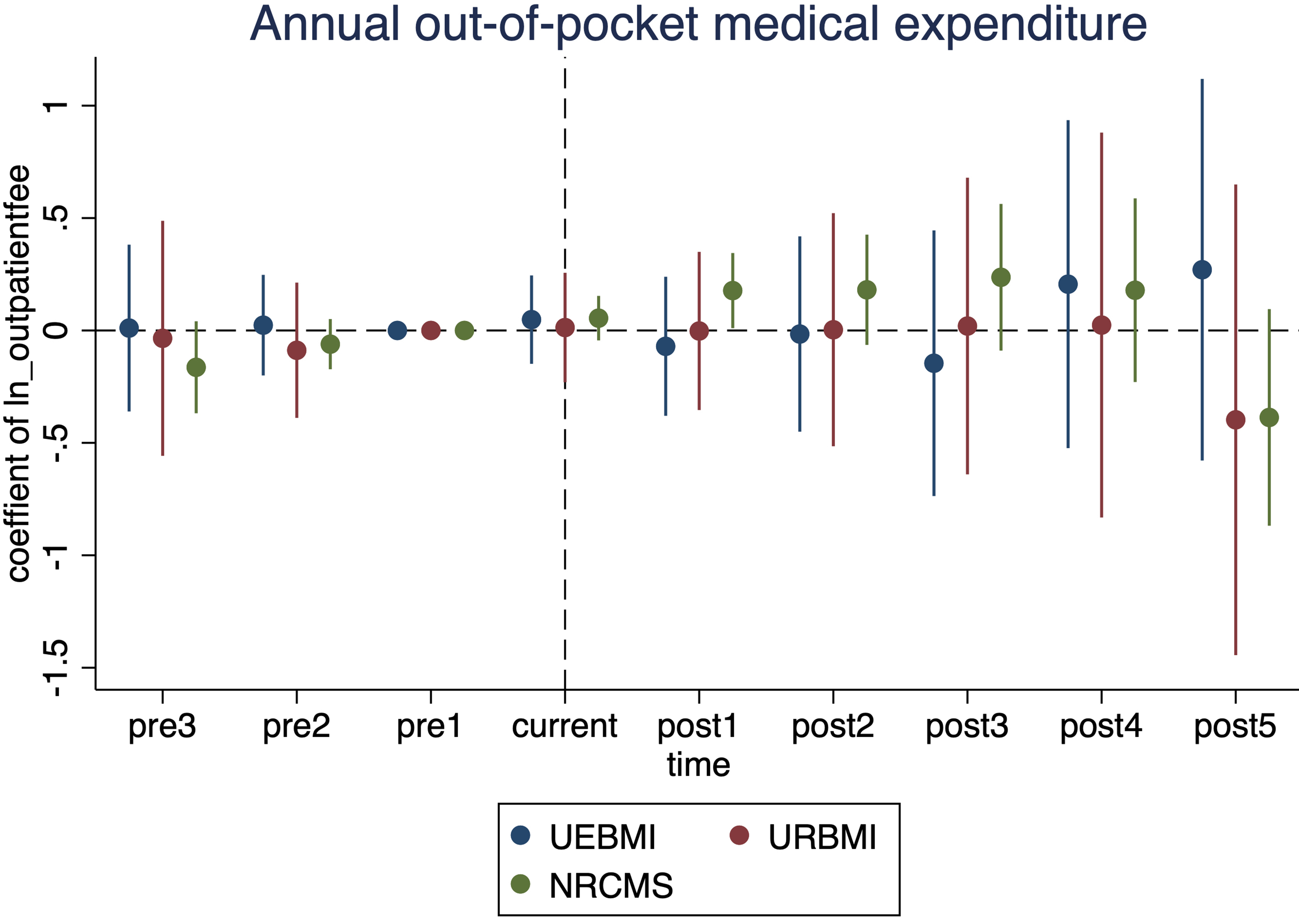

Supplement: Supplementary file 6 [file Image_5.JPEG]

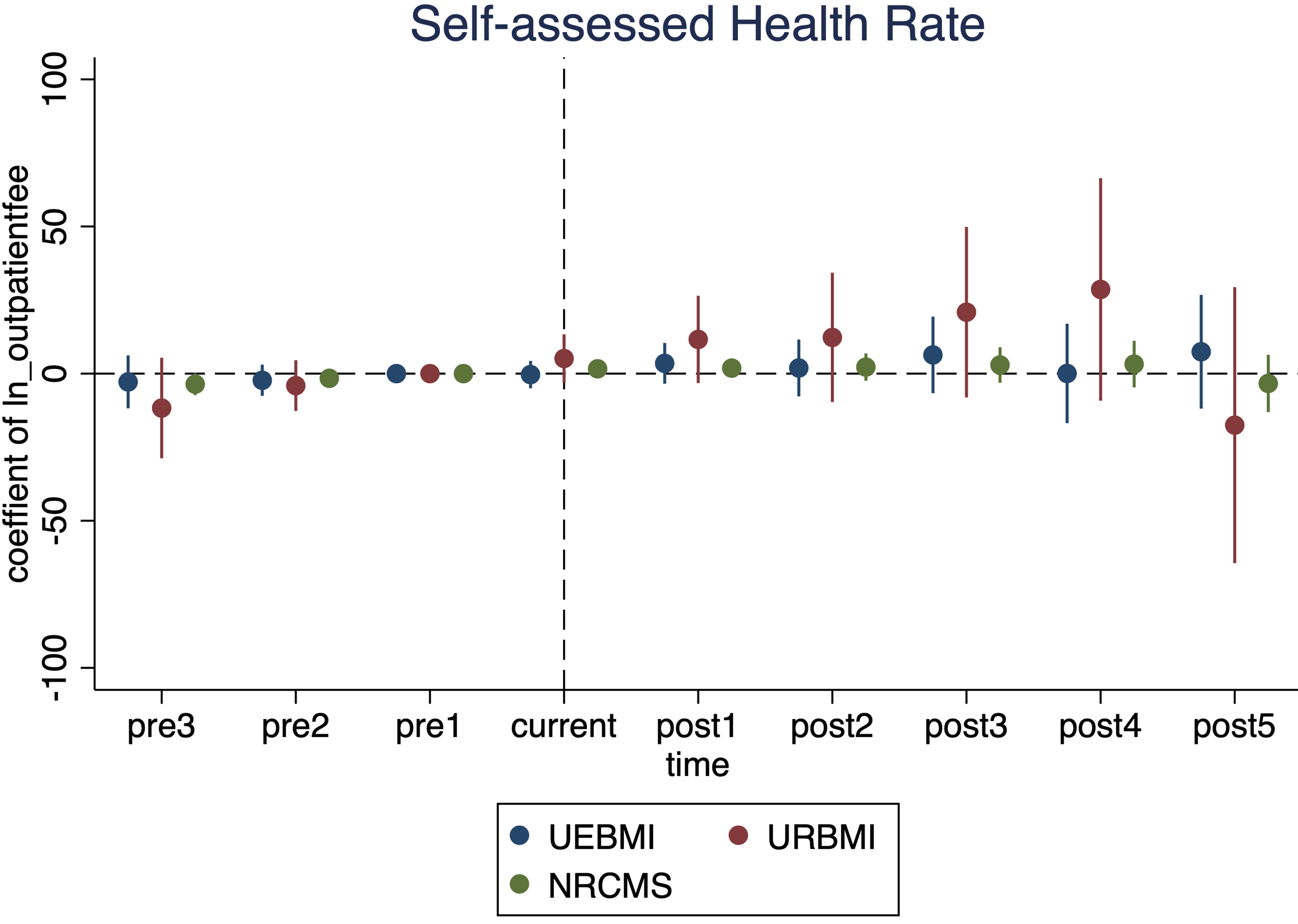

Supplement: Supplementary file 7 [file Image_6.JPEG]
